# Prevention Strategies in Endometrial Carcinoma

**DOI:** 10.1007/s11912-018-0747-1

**Published:** 2018-11-13

**Authors:** Michelle L. MacKintosh, Emma J. Crosbie

**Affiliations:** 1 Department of Obstetrics and Gynaecology, St Mary’s Hospital, Manchester Academic Health Science Centre, Manchester University NHS Foundation Trust, Manchester, UK; 2 Gynaecological Oncology Research Group, NIHR Manchester Biomedical Research Centre, Division of Cancer Sciences, School of Medical Sciences, Faculty of Biology, Medicine and Health, University of Manchester, 5th Floor Research, St Mary’s Hospital, Oxford Road, Manchester, M13 9WL UK

**Keywords:** Endometrial cancer, Obesity, Risk prediction, Prevention strategies, Weight loss, Levonorgestrel intrauterine system (LNG-IUS)

## Abstract

**Purpose of the Review:**

To assess the most recent high-quality evidence for endometrial cancer prevention strategies.

**Recent Findings:**

- Obesity is an established risk factor for endometrial cancer.
- Weight cycling and weight gain in middle age are risk factors for endometrial cancer.
- Bariatric surgery reduces the risk of endometrial cancer by up to 81% in obese women who attain and maintain a normal weight.
- Combined oral contraceptives provide durable protection against endometrial cancer for 30 years or more.
- Ever use of the levonorgestrel intrauterine system (LNG-IUS) and inert intrauterine devices reduce endometrial cancer risk.
- The first oestrogen-based non-progestin HRT for non-hysterectomised women that contains estradiol and bazedoxifene has an effective protective effect on endometrium.
- Bisphosphonates reduce endometrial cancer risk.

**Summary:**

Weight loss and LNG-IUS would seem to be an effective strategy for preventing the development of obesity-driven endometrial cancer in the highest risk women. Future research may identify other safe and effective chemoprevention interventions, such as aspirin, bisphosphonates or metformin.

## Introduction

The incidence of endometrial cancer continues to rise unabated. Over the past 20 years, the incidence has risen by more than 50%. In the UK alone, more than 9000 new cases are diagnosed each year and it is responsible for the deaths of more than 2000 women. The incidence in women under 50 increased by 2% every year between 1992 and 2012 [1].

An ageing population, changing patterns of hysterectomy use and tamoxifen therapy may all contribute to these trends, but the overwhelming culprit is undoubtedly the obesity epidemic. Across Europe, it has been estimated that 60% of endometrial cancer cases may be due to excess weight.

**Fig. 1:**
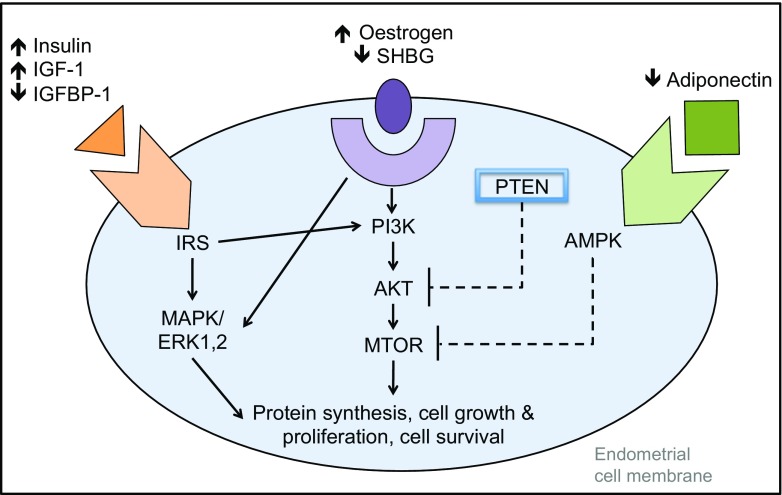
An overview of signal transduction pathways dysregulated in endometrial carcinogenesis

Most endometrial cancers are what Bokhman termed type 1 endometrial cancers, where low-grade cancer develops in a hyperplastic endometrium, often on a background of obesity or diabetes [2]. The biological mechanisms driving type 1 endometrial carcinogenesis are incompletely understood, but adipose-derived oestrogen, unopposed by progesterone in obese postmenopausal women, is the best-supported hypothesis [3]. Obesity per se is not the whole story, however; insulin resistance, systemic inflammation and genetic predisposition all contribute to susceptibility, providing opportunities for targeted prevention strategies. Other risk factors such as tamoxifen, nulliparity, unopposed oestrogen therapy and polycystic ovary syndrome (PCOS) are well described. Many of the recognised risk factors interact with key, pro-proliferative, signal transduction pathways (Fig. 1).

Worldwide, the prevalence of obesity (body mass index, BMI > 30 kg/m^2^) has doubled in the last three decades; each year, 2.8 million people around the world die as a result of being overweight or obese. Obesity accounts for 44% of the disease burden of diabetes and 23% of that of ischaemic heart disease [4].

Despite improving survival rates, deaths from endometrial cancer have increased by almost 20% in the last decade. Whilst across all stages 5-year survival reaches 79%, higher BMI is associated with increased all-cause mortality (per 10% increase in BMI OR 1.09; 95% CI 1.03–1.16), and disproportionate treatment-related morbidity [5].

## Prevention in High-Risk Groups

### Lynch Syndrome

Whilst the majority of endometrial cancer is sporadic, at least 3–5% of new diagnoses are made in women with a familial predisposition. The most common cause of this is Lynch syndrome (previously known as hereditary non-polyposis colorectal cancer, HNPCC), which is a highly penetrant autosomal dominant inherited predisposition to cancers of the endometrium, bowel, ovary and prostate, amongst others. In women with Lynch syndrome, the lifetime risk of endometrial cancer may be as high as 70%, compared with a lifetime risk of 2–3% in the general population, and often precedes a colorectal cancer diagnosis by approximately 10 years [6]. Identification of families with Lynch syndrome allows quantification of cancer risk and access to cancer surveillance programmes and could prevent cancers in other family members (Table 1) [7].

**Table 1:** Summary table of the main risk factors for endometrial cancer and existing and potential prevention strategies to minimise this risk

Risk factor	Effect on endometrial cancer risk	Proposed mechanism	Proven methods of prevention	Potential methods of prevention
Lynch syndrome	Lifetime risk 70%, cf. 2–3% in general population	Mutations in DNA mismatch repair genes	Risk-reducing surgery	Aspirin
Tamoxifen	Postmenopausal RR 4.01 (95% CI 1.7–10.9)	Oestrogenic effects on endometrium	Low threshold to investigate abnormal bleeding	LNG-IUS
PCOS	Lifetime risk 9% OR 2.89	Insulin resistance Anovulatory cycles	Induce regular withdrawal bleeds	Weight reduction Metformin hormonal contraception
Obesity	RR 1.59 per 5 kg/m^2^ increase in BMI	Activation of pro-proliferative pathways (Fig. 1) Anovulatory cycles	Bariatric surgery Physical activity	Non-surgical weight loss LNG-IUS
Diabetes	RR 1.42–4.1	Activation of pro-proliferative pathways	Bariatric surgery	Modulation of insulin resistance

Risk-reducing surgery, in the form of hysterectomy and bilateral salpingo-oophorectomy when family complete, is well established in the primary prevention of Lynch syndrome–associated endometrial and ovarian cancers (women with Lynch syndrome have a 10–12% lifetime risk of ovarian cancer) [8, 9]. No evidence of a reduction in mortality has been demonstrated with gynaecological cancer surveillance in Lynch syndrome [10, 11]. Whilst aspirin use has demonstrated a reduction in risk for colorectal cancer, the same may not be true for endometrial cancer and more research is needed. In their primary prevention trial, Burn et al. found an unadjusted non-statistically significant reduction in endometrial cancer risk with 600 mg aspirin once daily for up to 4 years, although it was not sufficiently powered for endometrial cancer endpoints [2].

Biomarker studies have shown that combined oral contraceptives and synthetic progestin reduce endometrial proliferation, in women with Lynch syndrome. However, it is unknown whether hormonal contraceptives are as effective for chemoprevention in Lynch syndrome as they are in the general population [12].

### Tamoxifen Use

Tamoxifen is a selective oestrogen receptor modulator (SERM) used for the prevention and adjuvant treatment of breast cancer. It has been associated with increased endometrial polyps and hyperplasia, and an increased relative risk of endometrial cancer in postmenopausal women of 4.01 (95% CI 1.7–10.9). There is no endometrial surveillance programme in place for tamoxifen users, but women and clinicians should be aware of the risks [13], and investigation of vaginal bleeding should include hysteroscopy as well as endometrial biopsy and ultrasound scan, as sensitivity and specificity of transvaginal ultrasound are low in this group. The levonorgestrel intrauterine system (LNG-IUS) has been shown to reduce the development of endometrial polyps in tamoxifen users but no convincing effect on endometrial cancer risk has yet been demonstrated [14].

### PCOS

PCOS affects 6–8% of women of reproductive age. Women with PCOS are overrepresented in young endometrial cancer patients, and a meta-analysis has estimated the lifetime risk of endometrial cancer in women with PCOS to be in the region of 9% (odds ratio (OR) 2.89), the increase likely driven by a toxic combination of insulin resistance and anovulatory cycles leading to oligo or amenorrhoea. For this reason, a crucial aspect of PCOS management is induction of regular withdrawal bleeds, in an attempt to reduce the risk of developing endometrial hyperplasia and cancer.

Whilst diet and weight reduction have been shown to improve the clinical manifestations of PCOS such as insulin resistance and reproductive manifestations, there is no evidence it reduces the risk of developing endometrial cancer [15]. Based on the presumption that insulin resistance is a key player in the pathogenesis of PCOS, metformin is being increasingly explored to ameliorate symptoms such as infertility and oligomenorrhoea. In vitro metformin has been shown to exert anti-tumour effects, but despite the promise of non-randomised clinical trials [16], there is no RCT evidence to support a role of metformin in endometrial cancer prevention in women with PCOS [17].

## Prevention in the General Population

### Weight and Physical Activity

The World Cancer Research Fund has concluded that the risk of endometrial cancer is reduced by moderate physical activity and maintaining a healthy weight [18]. Whilst obesity is a well-established risk factor for multiple types of cancer, the association is seen most strongly in endometrial cancer. Excess weight has been shown to have a non-linear, dose-dependent relationship with endometrial cancer risk [19, 20]. Every 5-kg/m^2^ increase in BMI increases the relative risk (RR) by 1.59, and at a BMI > 42 kg/m^2^, RR 9.11 (95% CI 7.26–11.51). Risk is higher in women who have never used hormone replacement therapy (HRT) (RR 20.7; 95% CI 8.28–51.84) [21].

Lifestyle modification can result in 4–6% weight reduction over 2 to 4 years, and anti-obesity drugs can lead to 7–10% weight reduction; however, only bariatric surgery produces significant and, crucially, durable results [22, 23]. Bariatric surgery has been shown to reduce cancer risk in women, particularly postmenopausal breast and endometrial cancers. A retrospective cohort study of more than 100,000 bariatric surgery patients in the USA estimated a 77–81% reduction in endometrial cancer risk associated with bariatric surgery (RR 0.29, 95% CI 0.26–0.32), and an even lower risk in women who attained and maintained normal weight post-bariatric surgery compared with those who remained overweight or obese (RR 0.19, 95% CI 0.17–0.22 compared with RR 0.48, 95% CI 0.43–0.55, respectively) [24].

Over 18-year median follow-up, the prospective, non-randomised SOS Study has demonstrated that bariatric surgery significantly reduces the risk of endometrial cancer (hazard ratio (HR) 0.56, 95% CI 0.35–0.89, *p* = 0.014) in over 2800 women who underwent surgical or conventional weight management treatment. Other cancers were also reduced after bariatric surgery and the number needed to treat (NNT) to prevent one cancer over 10 years with bariatric surgery was 31 [25•].

Studies continue to show that as well as excess body weight being a risk factor for endometrial cancer, so too is adult weight gain, weight cycling, the duration of overweight/obesity and possibly being overweight in childhood/at age 18 [26, 27•, 28–30]. In the NOWAC study, a 5-kg weight gain was significantly associated with increased risk of postmenopausal endometrial cancer, with a dose–response relationship with increasing weight gain, emphasising the importance of maintaining a stable weight in “middle adulthood” [28]. Weight gain and weight cycling have also been shown to be significantly associated with the development of postmenopausal endometrial cancer in the Women’s Health Initiative Observational Study, of 80,943 women including 788 cases of endometrial cancer over 20 years of follow-up [27•]. Weight cycling four to six times increased risk of endometrial cancer by 38% compared with weight-stable women.

There is now convincing evidence that women with higher physical activity levels have a lower risk of endometrial cancer than women with the lowest activity levels, in particular overweight or obese women with high activity levels (BMI < 25 RR 0.97; 95% CI 0.84–1.13 cf. BMI ≥ 25 RR 0.69; 95% CI 0.52–0.91) [31, 32•]. Studies of physical activity are limited by the activity being self-reported and more difficult to ascribe a quantitative value to, and heavily intertwined with, other confounding risk factors such as adiposity.

Arthur et al. modelled a Healthy Lifestyle Index (HLI) in 3185 women and reported that for every unit increase in HLI, there was a 5% reduction in endometrial cancer risk. When they directly compared the low vs. high HLI groups, there was a 46% reduction in risk [33•].

### Diabetes and Insulin Resistance

Diabetes may increase the risk of endometrial cancer; however, meta-analyses are frequently plagued by the confounders of inactivity and obesity, as alternative or additional endometrial cancer risk factors. After multivariate adjustment, studies have estimated the increase in relative risk to be in the region of 1.42 to 4.1 [34].

An umbrella review in 2015 supported an association between type 2 diabetes and endometrial cancer. It examined 8174 cases, found no significant bias within the studies, and reported Summary Random Effects estimates of 1.97 (95% CI 1.71–2.27), and 95% prediction intervals of 1.23 to 3.16 [35]. Sacerdote et al. published the results of their meta-analysis which supported an association between endometrial cancer and diabetes (RR 1.65, 95% CI 1.5–1.81), although high heterogeneity between studies was found [36].

It has not been proven that modulation of insulin resistance is an effective mechanism for preventing EC [19, 37]. Many studies assess surrogate markers rather than clinical end points, and a definitive chemoprevention study is unlikely as it would require huge numbers and many years of follow-up. Randomised biomarker studies have so far failed to demonstrate a reduction in endometrial proliferation with 2–16 weeks of metformin treatment [38, 39]. Similarly, metformin has not been shown to reduce endometrial cancer risk (OR 1.05, 95% CI 0.82–1.35, *p* = 0.7), but may improve risk of recurrence or overall survival [40].

### Hormonal Treatment

Long-term follow-up data provides convincing evidence that use of combined oral contraceptives (COC) is associated with a significant and enduring reduction in the lifetime risk of endometrial cancer. Iversen et al. demonstrate that the protective effects of COC persist for at least 30 years, in this updated follow-up study of users who were recruited between 1968 and 1969 [41•]. The faculty of Sexual and Reproductive Health advises that at a BMI > 35 kg/m^2^, the risks of COC are likely to outweigh the benefits (UKMEC 3 recommendation), which precludes its use as chemoprevention in the most obese women [42].

Oral, injectable and intrauterine progestin use has been shown to reduce the risk of endometrial cancer. Data from the Finnish Cancer Registry showed a standardised incidence ratio of 0.46 for endometrial cancer in users of the LNG-IUS. The NOWAC study was a Norwegian population-based prospective cohort study. Median follow-up was 12.5 years; 9% of the cohort reported LNG-IUS use during or prior to 1998–2007. After adjusting for BMI, activity, age at start of follow-up, combined oral contraceptive use, menopausal status and parity ever-users of LNG-IUS had RR of endometrial cancer of 0.34 (95% CI 0.18–0.65) compared with never-users [43•].

Almost 25 years ago, the harmful effects of unopposed oestrogen HRT in women with a uterus were apparent. Sequential HRT has also been shown to increase the risk of endometrial cancer, with risk being inversely proportional to the number of days progestin is given for. Continuous combination has not been shown to increase endometrial cancer risk and may even reduce it, presumably because of the protective effects of progesterone on the endometrium [44].

Recent years have seen the emergence of the first oestrogen-based, progestin-free oral menopausal HRT for non-hysterectomised women. Conjugated oestrogens with the SERM bazedoxifene (CE/BZA) minimise estrogenic effects on endometrium and breast whilst effectively addressing menopausal symptoms and protecting against osteoporosis. A similar randomised, double-blind study of 17β-estradiol/raloxifene did not provide adequate endometrial protection. CE/BZA has been studied in five RCTs involving more than 7500 women and no increase in endometrial hyperplasia was found. It may be an option for women who poorly tolerate the side effects of progestins [45•].

### Bisphosphonates

Preclinical and animal model studies have shown that bisphosphonates have anti-tumour effects, in part through induction of apoptosis and inhibition of proliferation and angiogenesis. They have been shown to affect the growth and metastasis of gynaecological cancers in cell lines and animal models. A meta-analysis of cohort and case control studies demonstrated a statistically significant endometrial cancer risk reduction with 1–3 years of bisphosphonate use in 6499 endometrial cancer cases from 226,560 participants. With 3 or more years of bisphosphonate use, a 56% reduction in endometrial cancer risk was seen (pooled RR 0.44; 95% CI 0.28–0.7) [46•].

### Aspirin

In their 2005 randomised controlled primary prevention trial of 39,876 women in the Women’s Health Study, Cook et al. found no association between low-dose aspirin use (100 mg/alternate days cf. placebo) and endometrial cancer risk (RR 1.22, 95% CI 0.94–1.58, *p* = 0.14) [47]. A 2016 meta-analysis of cohort and case control studies did suggest a small-to-modest protective effect with aspirin use, and a weak effect with regular NSAID use, from statistically non-significant pooled risk estimates [48]. The results should be interpreted with caution; only cohort and case control studies were included, and doses and duration of aspirin use varied widely between the studies included. A 2018 meta-analysis by Qiao and Yang et al. also found an inverse association between aspirin use and endometrial cancer risk (RR 0.92, 95% CI 0.85–0.99) from 8410 endometrial cancer cases in six case control studies, and 3127 endometrial cancer cases in eight cohort studies. Again, it is unclear what dose of aspirin participants were taking [49].

### Reproductive

Continued efforts to promote breastfeeding may help to reduce endometrial cancer risk in the general population. In their meta-analysis, Jordan et al. demonstrated an 11% reduction in endometrial cancer risk in women who had breastfed (pooled OR 0.89; 95% CI 0.81–0.98) comparing 8981 endometrial cancer cases with 17,241 controls from cohort and case control studies [50].

Felix et al. presented a pooled analysis of individual level data from 18 epidemiological studies, which was the largest investigation of IUD use and EC risk to date (8801 cases and 15,357 controls). Pooled OR for ever-use of IUD was 0.81 (95% CI 0.74 to 0.9) compared with never-use with the inverse association being strongest in users of inert IUDs, and weaker in users of copper, hormonal or a combination of types of IUDs. Nulliparous IUD users gained more benefit than parous IUD users. Proposed mechanisms of action by which IUDs reduce endometrial cancer risk are increased decidual loss, alterations in hormone receptor expression and stimulation of an inflammatory microenvironment in the uterus [51].

### Dietary

#### Soy

A meta-analysis of 13 studies (no RCTs) demonstrated a weak inverse relationship between high isoflavone (soy) consumption and endometrial cancer risk [52]. Novasoy and genistein have been shown to inhibit proliferation by reducing oestrogen receptor (ER) alpha expression and interacting with the pro-proliferative AKT/MTOR/MAPK pathway and have been suggested as potential therapeutic agents [53].

#### Coffee

The largest meta-analysis in the literature, including 1.4 million participants and 10,100 cases of endometrial cancer, with follow-up of 11 to 20 years concluded there was a dose-dependent reduction in endometrial cancer risk with coffee intake, stronger but not limited to caffeinated coffee. One cup of caffeinated coffee per day was associated with a 7% reduction in endometrial cancer risk (RR 0.93, 95% CI 0.89–0.97), compared with a 4% risk reduction with one cup of decaffeinated coffee/day (RR 0.96, 95% CI 0.92–0.99) [54•].

Merritt et al. conducted a nutrient-wide association study (NWAS) on data from 1303 endometrial cancer cases from the EPIC study and 1531 endometrial cancer cases from the Nurses Health Studies and confirmed an inverse association between coffee intake and endometrial cancer risk [55].

Several factors are thought to contribute to the protective effect of coffee. Caffeine is associated with levels of sex hormone-binding globulin (SHBG) and, as a consequence, levels of bioavailable oestrogen and testosterone. Coffee contains antioxidants and chlorogenic acid, which may inhibit glucose absorption. Coffee has been shown to have an inverse relationship with plasma C peptide levels and the risk of diabetes mellitus [56].

#### Tea

A meta-analysis by Zhou et al., which included six case control or cohort studies, not all of which were controlled for physical activity, found a reduced risk of endometrial cancer with higher intake of green tea. One cup per day was associated with an 11% risk reduction in a dose–response analysis (RR 0.89; 95% CI 0.84–0.94). No protective effect was seen with black tea. Green tea has higher levels of catechins than black tea, such as epigallocatechin gallate, which has been found to induce apoptosis and cell cycle arrest, and inhibit oestrogen-induced activation of endometrial cells.

## Our Approach

The main focus of our research is the prevention and early detection of endometrial cancer. These themes ranked as the most important endometrial cancer research priorities for patients, carers and healthcare professionals in our award-winning James Lind Alliance Priority Setting Partnership [57]. We found a high prevalence of occult endometrial abnormality in obese women undergoing bariatric surgery, and witnessed their reversal to healthy endometrium after bariatric surgery-induced weight loss in some cases [58]. Based on these findings, we have made local arrangements to facilitate the provision of bariatric surgery to obese women diagnosed with an obesity-related precancerous endometrial lesion and have ongoing work exploring non-surgical treatments for low-grade early-stage endometrial cancers in selected patients.

Our aim is to identify a high-risk endometrial tissue signature that predicts those women at greatest risk of developing endometrial cancer to target for prevention measures (such as weight loss, LNG-IUS ± chemoprevention agents). Our theoretical risk prediction model will facilitate the identification of women for stratified prevention interventions and ongoing work seeks to validate our model in large UK populations [59]. Such interventions may also be useful in the post-treatment setting, to reduce the risk of cancer recurrence, although there is currently no evidence to support this [60]. Achieving and maintaining weight loss is likely to have health benefits beyond the endometrium in obese postmenopausal women, for example by reducing the risk of type 2 diabetes mellitus [61] and cardiovascular events [62].

## Future Research

Future research must target the gaps in our knowledge. Can we identify those at highest risk of developing endometrial cancer and how best can we prevent it? Well-designed observational and interventional studies may address the questions of aspirin, NSAID and other potential chemoprevention agents. The degree of benefit will need to be weighed against the potential adverse effects of such drugs.

## Conclusions

For now, it would seem that prevention strategies should focus on minimising risk through weight reduction and/or stability, improving access to bariatric surgery, and by educating women and healthcare professionals about the importance of weight and activity levels on their risk of type 1 endometrial cancer. The LNG-IUS is an effective tool for women at highest risk.

Future research may provide us with the justification to use aspirin, metformin or bisphosphonates for chemoprevention but at present the evidence for either their efficacy or their long-term safety in high-risk women is lacking.

Use of the Healthy Lifestyle Index may help women take control of minimising their personal risk and help healthcare professionals quantify risks for patients. Work must continue in identifying a high-risk molecular/phenotypic signature for targeted prevention, and efforts to identify women with Lynch syndrome must continue.
